# Time dependent effects of adjuvant tamoxifen therapy on cerebrovascular disease: results from a randomised trial

**DOI:** 10.1038/bjc.2011.45

**Published:** 2011-02-22

**Authors:** J Rosell, B Nordenskjöld, N-O Bengtsson, T Fornander, T Hatschek, H Lindman, P-O Malmström, A Wallgren, O Stål, J Carstensen

**Affiliations:** 1 Oncologic center, Linköping University Hospital, S-581 85 Linköping, Sweden; 2 Division of Oncology, Department of Clinical and Experimental Medicine, Linköping University, S-581 83 Linköping, Sweden; 3 Department of Oncology, Umeå University Hospital, S-901 85 Umeå, Sweden; 4 Department of Oncology, Karolinska University Hospital, S-171 76 Stockholm, Sweden; 5 Department of Oncology, Uppsala University Hospital, S-751 85 Uppsala, Sweden; 6 Department of Oncology, Lund University Hospital, S-221 85 Lund, Sweden; 7 Department of Oncology, Sahlgrenska University Hospital, S-413 45 Göteborg, Sweden; 8 Department of Health and Society, Linköping University, S-581 83 Linköping, Sweden

**Keywords:** breast cancer, tamoxifen, adjuvant treatment, adverse events, cerebrovascular disease

## Abstract

**Background:**

Tamoxifen has been associated with an increased risk of stroke. There is, however, little information on the effect in the post-treatment period. Using data from the Swedish Breast Cancer Group adjuvant trial of 5 *vs* 2 years of tamoxifen treatment, we now report both short-term and long-term effects on morbidity as well as mortality because of cerebrovascular disease.

**Methods:**

Data from the Swedish National Hospital Discharge Registry combined with information from the Swedish Cause of Death Registry was used to define events of disease. Hazard ratios (HRs) were estimated using Cox regression.

**Results:**

Comparing patients randomised to 5 years of tamoxifen with patients randomised to 2 years of tamoxifen, the incidence of cerebrovascular diseases was increased (HR 1.70, 95% CI 1.05–2.75) during the active treatment phase and reduced after the active treatment period (HR 0.78, 95% CI 0.63–0.96), and the difference in HR between the two time-periods was significant (*P*=0.0033). The mortality from cerebrovascular diseases was increased during the treatment period (HR 3.18, 95% CI 1.03–9.87) and decreased during the post-treatment period (HR 0.60, 95% CI 0.40–0.90) with a significant difference in HR between the two periods of follow-up (*P*=0.0066). Similar results were seen for subgroups of cerebrovascular diseases, such as stroke and ischaemic stroke.

**Conclusion:**

In an adjuvant setting, tamoxifen was associated with an increased risk of cerebrovascular disease during treatment, but a decreased risk in the post-treatment period.

In 1996 and 2005, we reported from a randomised tamoxifen trial in postmenopausal women with early-stage breast cancer younger than 75 years of age at surgery (Swedish Breast Cancer Cooperative Group, 1996; Nordenskjöld *et al*, 2005). The all-cause mortality, breast cancer-specific mortality and coronary heart disease mortality were significantly reduced in the 5-year group, compared with the 2-year group. We also saw a tendency to a decreased mortality rate from cerebrovascular diseases in the 5-year group compared with the 2-year group (hazard ratio (HR) 0.81, 95% CI 0.53–1.23). The most recent EBCTG overview of randomised trials shows a non-significant excess of mortality from stroke in the group receiving about 5 years of adjuvant tamoxifen *vs* control (Early Breast Cancer Trialists’ Collaborative Group, 2005). Several meta-analyses of breast cancer risk reduction and treatment trials showed that tamoxifen is associated with an increased risk of stroke (Braithwaite *et al*, 2003; Bushnell and Goldstein, 2004; Nelson *et al*, 2009). Many of the studies contributing to these meta-analyses, however, have a limited follow-up time and there is little information on the period after the termination of tamoxifen treatment. With longer follow-up and with added data on hospitalisations, we now report both short-term and long-term effects on the incidence of cerebrovascular disease as well as mortality from cerebrovascular diseases in the Swedish Hospital Discharge Registry (HDR) and in the Swedish Cause of Death Registry.

## Materials and methods

The design and performance of the trial were described in detail in the first report (Swedish Breast Cancer Cooperative Group, 1996). The study included data from five of the six Swedish health care regions; two of which used 20-mg daily doses of tamoxifen and three of which used 40-mg. From 1983 to 1992, a total of 4610 patients were entered in the trial; of whom 4150 remained alive and recurrence free at 2 years, and could thus contribute meaningful information to the comparison of outcomes associated with prolonged tamoxifen treatment. Of these 4150 patients, 32% were <60 years of age at surgery, 12% older than 70 years, and 53% were lymph-node positive. In total, 40% of the patients received 20-mg daily doses of tamoxifen and 60% were given 40-mg daily doses of tamoxifen. Among the patients who have breast cancer as an underlying cause of death and no distant recurrence, we have updated with new data on distant recurrences and used the date of main diagnosis of breast cancer in HDR to substitute missing data of distant recurrences. These additional data resulted in a reduction of the number of patients from 4175 in our previous report (Nordenskjöld *et al*, 2005) to 4150 patients.

In the analysis, the start date was 2 years after date of surgery and patients were censored at date of death. Furthermore, to avoid possible confounding effects of recurrence of breast cancer and consequent treatment, each patient was considered to be at risk for the analysed events up to, but not beyond, that of any recurrence or contralateral breast cancer. Cox proportional hazards modelling stratified by trial centre was used to estimate hazard ratios and confidence intervals. Cumulative incidence of cerebrovascular diseases was estimated by use of life-table methods. To test for differences in HR between the follow-up periods, an interaction term between treatment group and time period was added to the Cox model. The effect of doses of tamoxifen (20 *vs* 40 mg) on cerebrovascular disease, during and after active treatment, respectively, were checked, and no significant difference in HR between the doses were detected. Also the interaction effect of treatment and age at surgery (dichotomised at the median age; 63 years) on cerebrovascular disease was checked, during and after active treatment, and none of the interactions was significant.

## Results

**Figure 1:**
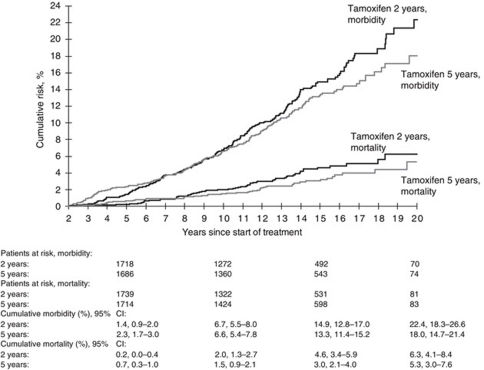
Cumulative morbidity and mortality from cerebrovascular diseases among patients randomly assigned to 2 years (*n*=2114) or 5 years (*n*=2036) of adjuvant tamoxifen therapy. Patients at risk and cumulative risk estimates with 95% confidence intervals (CI) are given at 5, 10, 15 and 20 years after start of treatment.

An intention-to-treat analysis revealed that 5 years of tamoxifen treatment compared with 2 years of tamoxifen treatment was associated with an increased risk of cerebrovascular diseases (HR 1.70, 95% CI 1.05–2.75) during the active treatment phase and a reduced risk after the active treatment period (HR 0.78, 95% CI 0.63–0.96) (Table 1 and Figure 1). The difference in HR associated with cerebrovascular diseases between the two periods was statistically significant (*P*=0.0033). We also saw significant differences in HR between the periods of follow-up for stroke (*P*=0.0056) and ischaemic stroke (*P*=0.014), and similar tendencies for haemorrhagic stroke, other and unspecified cerebrovascular diseases and for TIA (Table 1). The mortality from cerebrovascular diseases was increased during the treatment period (HR 3.18, 95% CI 1.03–9.87) and decreased during the post-treatment period (HR 0.60, 95% CI 0.40–0.90), with a significant difference in HR between the periods of follow-up (*P*=0.0066). Similar significant differences in HR were obtained when the data were not censored for contralateral breast cancer or any recurrence (data not shown).

## Discussion

In the analysis, the date of main diagnosis from HDR or date of death was used. We have not reviewed the diagnoses in this study, but an earlier validation study showed a good precision of main diagnosis in HDR for cerebrovascular diseases with sensitivity 95.7% and specificity 99.5% (Nilsson *et al*, 1994). After a median follow-up after surgery of 12.1 years (range: 2.0–23.9 years), we found a time-dependent effect of tamoxifen on the incidence of cerebrovascular diseases. Our data confirm previous findings of increased risk of cerebrovascular diseases in patients receiving postoperative tamoxifen therapy (Braithwaite *et al*, 2003). There are very few studies about the effect of tamoxifen on the incidence of cerebrovascular diseases after the active treatment period. Two studies have not been able to show that there is a decreased risk of cerebrovascular disease during the post-treatment period (McDonald *et al*, 1995; Cuzick *et al*, 2007). However, the women were both pre- and postmenopausal and the number of events was quite small in the treatment arms after tamoxifen was stopped. Interestingly, the ATAC trial finds an excess risk of cerebrovascular accidents during treatment in the tamoxifen arm compared with the anastrozole arm (34 *vs* 20 events), but no difference off treatment (20 *vs* 22 events) (ATAC Trialists’ Group, 2008).

Tamoxifen is associated with several of positive effects, for example, lipid-lowering properties by decreasing total and low-density lipoprotein cholesterol, and decreasing the levels of C-reactive protein (CRP), which are known risk factors for cardiovascular diseases, especially among postmenopausal women (Cushman *et al*, 2001). Tamoxifen increases the risk of venous thromboembolism in women with breast cancer, but its relationship to stroke is uncertain. As a result of the pro-thrombotic properties, tamoxifen may be associated with cerebral venous thrombosis (Bushnell and Goldstein, 2004). Tamoxifen exerts oestrogenic effects in several human tissues. As compared with the data from randomised trials with oestrogen therapy (Magliano *et al*, 2006), our data with tamoxifen show similar effects on cerebrovascular disease during the active treatment phase. However, cerebrovascular mortality and morbidity in the oestrogen trials were not analysed for the long-term effects, which we have described in this report. The time-dependent effects of tamoxifen on cerebrovascular disease in the present study thus might be the result of a combination of positive and negative effects of the treatment. However, as the biological mechanisms are poorly understood, the long-terms effects of tamoxifen should be interpreted with caution.

In summary, this trial showed a time-dependent effect of postoperative tamoxifen treatment, with a significantly increased risk of morbidity and mortality from cerebrovascular diseases during the active treatment phase and significantly decreased risks after the active treatment period. The difference in HR between the two follow-up periods was statistically significant for cerebrovascular diseases, as well as for mortality from cerebrovascular diseases. Also, for stroke and ischaemic stroke there was a statistically significant difference. These results stress the importance of obtaining long-term follow-up data from adjuvant trials also comparing tamoxifen with aromatase inhibitor, as well as from prevention trials comparing tamoxifen with other chemopreventive agents (Visvanathan *et al*, 2009). Our finding of a benefit of tamoxifen on cerebrovascular risk in the post-treatment phase is new and needs to be confirmed by more data from other studies.

## Figures and Tables

**Table 1:** Morbidity and mortality from cerebrovascular disease according to randomised treatment (5 *vs* 2 years of tamoxifen) for the entire follow-up (>2 years after start of treatment), during treatment (2–5 years) and after treatment (>5 years)

		**Number of events**				
	**Years since start of treatment**	**2-year group (*n*=2114)**	**5-year group (*n*=2036)**	**Hazard ratio (5 *vs* 2 years) (95% CI)**	***P*-value^a^**	***P* diff.**
*Morbidity*						
Cerebrovascular diseases (430–8)^b^	>2	213	198	0.89 (0.73–1.08)	0.23	
	2–5	27	44	1.70 (1.05–2.75)	0.030	
	>5	186	154	0.78 (0.63–0.96)	0.020	0.0033
Stroke (431, 433, 434, 436)^b^	>2	167	148	0.84 (0.67–1.05)	0.13	
	2–5	20	33	1.72 (0.99–3.00)	0.056	
	>5	147	115	0.73 (0.57–0.93)	0.011	0.0056
Ischaemic stroke (433, 434)^b^	>2	115	119	0.98 (0.76–1.27)	0.89	
	2–5	13	26	2.10 (1.08–4.09)	0.029	
	>5	102	93	0.85 (0.64–1.12)	0.25	0.014
Haemorrhagic stroke (431)^b^	>2	27	22	0.76 (0.43–1.33)	0.34	
	2–5	5	6	1.22 (0.37–3.99)	0.75	
	>5	22	16	0.66 (0.35–1.26)	0.21	0.37
Other and unspecified cerebrovascular	>2	76	79	0.99 (0.72–1.36)	0.97	
diseases (430, 432, 435, 437, 438)^b^	2–5	9	16	1.85 (0.82–4.19)	0.14	
	>5	67	63	0.88 (0.63–1.25)	0.48	0.10
TIA (435)^b^	>2	14	17	1.22 (0.60–2.47)	0.59	
	2–5	4	8	2.07 (0.62–6.86)	0.24	
	>5	10	9	0.88 (0.36–2.18)	0.79	0.27
						
*Mortality*						
Cerebrovascular diseases (430–8)^b^	>2	62	50	0.75 (0.52–1.10)	0.14	
	2–5	4	12	3.18 (1.03–9.87)	0.045	
	>5	58	38	0.60 (0.40–0.90)	0.014	0.0066

Abbreviations: CI=confidence interval; *P* diff.=*P*-value for difference in HR between the time-periods during and after treatment.

a Two-sided Wald test.

b Ninth International Classification of Diseases code numbers.
